# Age-dependence of electromagnetic power and heat deposition in near-surface tissues in emerging 5G bands

**DOI:** 10.1038/s41598-021-82458-z

**Published:** 2021-02-17

**Authors:** Giulia Sacco, Stefano Pisa, Maxim Zhadobov

**Affiliations:** 1 Department of Information Engineering, Electronics and Telecommunications, Sapienza University of Rome, Rome, 00184 Italy; 2 Univ Rennes, CNRS, IETR (Institut d’Électronique et des Technologies du numéRique) UMR 6164, 35000 Rennes, France

**Keywords:** Biomedical engineering, Biological physics

## Abstract

With the development of 5th generation (5G) mobile networks people of different ages will be exposed in the upper part of the microwave spectrum. From the perspective of non-ionizing radiation dosimetry, an accurate analysis of age-dependent electromagnetic power deposition and resulting heating is required. In this study, we evaluate the effect of age on exposure at 26 GHz and 60 GHz. A near-surface tissue model illuminated by a plane wave is used to asses the exposure considering both frequency-independent and frequency-dependent limits. The age-related variation of the skin thickness and tissue electromagnetic properties has been considered. Moreover, the blood flow decrease rate has been taken into account to assess the age-dependent heating. Our results demonstrate that the overall variations of the power density, specific absorption rate (SAR) and heating in the near-surface tissues are limited to about 10–15%. These variations are mainly due to the tissue permittivity and blood flow change with age. In contrast to the transmitted power density that increases with age, the peak SAR decreases at both frequencies. The peak steady-state heating increases from 5 to 70 years old by roughly 11% at 26 GHz and 13% at 60 GHz.

## Introduction

The increasing demand for high data rate mobile communications leads to fast development of the 5th generation (5G) cellular mobile networks^1,2^. Since the upper limit of the spectrum used for 5G networks has progressively shifted towards the millimeter waves (MMW), the roll-out of this technology will involve electromagnetic exposure at the world population scale to MMW frequencies, absent from the natural environmental electromagnetic spectrum^3,4^. Along with the massive transition to 5G technologies, more and more children and elderly people use wireless communication devices expanding the age range of exposed population. In this context, accurate dosimetry and understanding of power deposition mechanisms that depend on the physiological variations of the tissue properties with age are of uppermost importance.

Limits for human exposure to electromagnetic fields have been defined by major international organizations such as the International Commission on Non-ionizing Radiation Protection (ICNIRP) and the International Electrical and Electronics Engineers (IEEE)^5,6^. In the 6–300 GHz range, both guidelines limit the incident power density (IPD) for whole body exposure to ${10}\,{\hbox {W m}}^{-2}$ and 50 ${\hbox {W m}}^{-2}$ averaged over 30 min, for unrestricted environment/general public exposure and restricted environment/occupational exposure, respectively. For local exposures, frequency-dependent limits of IPD have been recently recommended, both by ICNIRP and IEEE. Specifically, the guidelines suggest $55 f_G^{-0.177}$ ($f_G$: frequency in GHz) for unrestricted environment/general public exposure while, for restricted environment/occupational exposure, the maximal IPD is set to $275f_G^{-0.177}$ and to $274.8f_G^{-0.177}$ by ICNIRP and IEEE, respectively^5,6^. In both guidelines, the IPD values should be averaged over 6 min over a surface of ${4}\,\hbox {cm}^{2}$. In addition, above 30 GHz, to account for a smaller beam diameter, the IPD, averaged on a area of 1 $\hbox {cm}^{2}$, is allowed to exceed the exposure limit by a factor of 2.

At MMW, the penetration depth in soft tissues (e.g. roughly 0.85 mm at 30 GHz and 0.5 mm at 60 GHz in cutaneous tissues) is small and the electromagnetic power absorption is mainly limited to skin^7,8^. Multilayer models of skin have been developed to characterize the energy absorption and resulting temperature elevation at MMW^9–12^. While these generic models are useful to asses MMW exposure, they do not account for the variability introduced by the skin ageing process. Therefore, it is fundamental to develop age-dependent models and compare exposure of children, adults and elderly people. At lower microwave frequencies, the difference in exposure levels between children and adults has already been investigated^13–15^. It has been found that, up to 5.6 GHz the SAR limits in children can be exceeded by 40–45% when the basic restrictions in adults are not exceeded^14,16^. However a more recent study^17^, using for the child models the standard dimensions specified by the Commission on Radiological Protection (ICRP) instead of the adult scaled ones, showed that the increase in the whole body SAR is not significant compared to the uncertainty expected in the numerical calculations.

To the best of our knowledge, no data on age dependence are available at MMW. The main goal of this study is to analyze how the age-varying physical parameters impact the power deposition and resulting heating in near-surface tissues. For the first time, this analysis is performed at 26 GHz and 60 GHz, frequencies upcoming for 5G and potentially for future generations. For the sake of brevity, in the rest of the paper 26 GHz, which is slightly below the lower limit of the MMW band, is also referred as MMW, since at this frequency the physical interactions with the human body are similar to those in the lower part of the MMW band.

## Materials and methods

### Near-surface tissue model

Above 6 GHz, skin absorbs most of the incident microwave energy due to a shallow penetration depth^18^. Skin is composed of two major layers: epidermis and dermis. The epidermis can in turn be divided in the stratum corneum (SC), the outer layer and the remaining viable epidermis. SC, with the thickness ranging from 10 to 20 $\upmu \hbox {m}$, contains a relatively small amount of water (typically $2\%$^18^) and has a negligible effect on MMW power absorption in skin. However, at some specific locations where SC is thicker (e.g. palms and soles of the feet, where it can reach 1.4 mm^19^), it impacts the power deposition above 15 GHz, by acting as a wave impedance matching layer therefore increasing the absorption^19^. The viable epidermis and dermis have almost the same water content and from the electromagnetic viewpoint can be considered as the same layer^18^. However, these layers have a different ability to dissipate heat, since only the dermis contains blood vessels.

**Figure 1:**
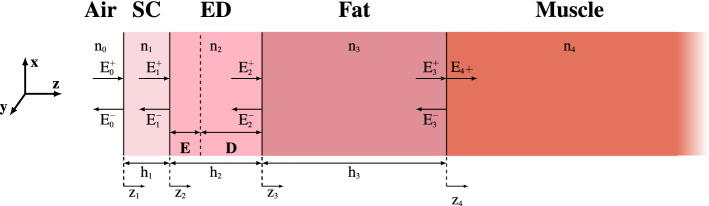
Four layer model employed for calculations of reflection, power deposition, and heating. The model includes SC, viable epidermis (E), dermis (D), fat, and muscle.

In this study, we use a multilayer model (Fig. 1) for the dosimetric assessment. The model is composed of four layers: SC, ED (viable epidermis + dermis), subcutaneous fat, and muscle. For the thermal analysis, ED is further divided in the viable epidermis and dermis layers (*E* and *D* in Fig. 1), to take into account the difference in blood perfusion. Although at MMW most of the electromagnetic power is deposited in skin, the underlying fat and muscle also contribute to the heating and therefore are included in the thermal model. The fat layer acts as a thermal insulator, whereas the muscle layer is significant to correctly describe the thermal diffusion^18^.

The tissue model is illuminated by a transverse electromagnetic (TEM) plane wave with normal incidence. The complex permittivity of the different layers is considered to be age-dependent according to the available literature data. Since at MMW most of the power is dissipated inside the skin layer, it is reasonable to assume that the entire power transmitted at the interface fat/muscle is dissipated inside the muscle layer.

### Principal ageing parameters

Human tissues evolve with age resulting in different electromagnetic energy absorption in the human body with age. For what concerns the electromagnetic power deposition, the two fundamental parameters to take into account are the water content, which directly influences the permittivity of tissues, and the thickness of different tissues. For evaluation of the temperature elevation also the variation of the blood flow with age is to be considered.

#### Thickness

**Figure 2:**
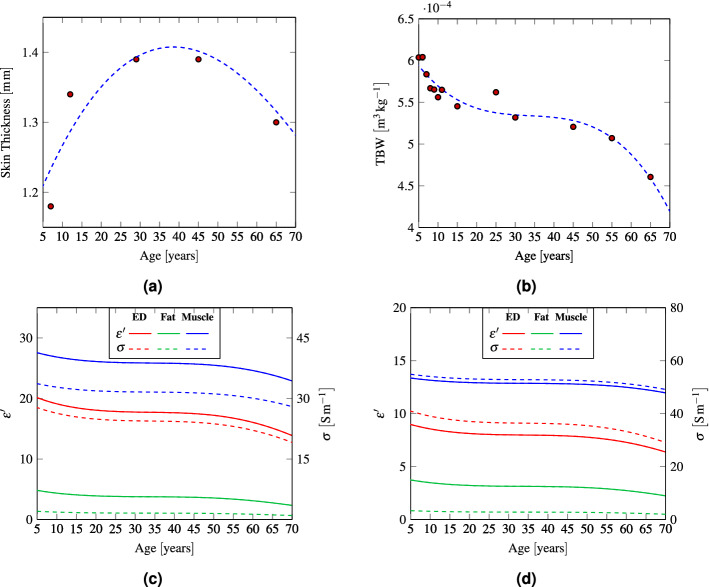
(**a**) Forearm skin thickness^23,30^ (**b**) TBW data^35,36^, permittivity $\varepsilon '$, and conductivity $\sigma$ as a function of age (**c**) at 26 GHz and (**d**) at 60 GHz.

Variations of the skin thickness (i.e., the physical and electrical length of the skin layer) may influence the electromagnetic power deposition and thermal diffusion. For the SC no or negligible changes with age have been found^20–22^ . Literature data on skin thickness indicate that overall skin thickens during youth^23^, while data on skin thickness during adulthood and elderly age are instead more difficult to interpret^24,25^. In fact, some authors found that the skin thickness remains constant in adulthood and decreases in the elderly age^26,27^. Other authors reported that, at specific body sites (e.g. face and buttock), skin thickens with age from adulthood to elderly age. The thickening with age of facial skin was explained by the elevated photo-exposition of this body region^28^, while the thickening of the buttock skin was attributed to a possible difference between extremities and axial skin^29^. In this study, we use the skin thickness reported for the dorsal forearm for children and adult males (Fig. 2a)^23,30^. These data have been chosen since they were measured by the same authors using the same technique (20 MHz ultrasound B scanner) and are in agreement with other results reported in the literature^28,31,32^.

#### Permittivity

Human tissues are composed of organic materials (that depends on the tissue) and water, whose amount changes with age. The complex permittivity for a tissue is expressed as $\varepsilon ^*=\varepsilon '-j\varepsilon ''$, where $\varepsilon '$ is the real part of the permittivity and $\varepsilon ''=\sigma /(2\pi f\varepsilon _0)$ is the imaginary part, depending on the conductivity $\sigma$, the frequency *f* and the free space permittivity $\varepsilon _0$. Since $\sigma /(\varepsilon '\varepsilon _0)$ does not depend on age^13^, for the sake of simplicity, the term $\varepsilon ''/\varepsilon '$, that depends only on frequency, will be referred with the subscript *A*, standing for adult ($\varepsilon ''_A/\varepsilon '_A$). The age variations of $\varepsilon '$, that rely on the water content, can be described by the Lichtenecker’s exponential law^13^, according to

1 $$\begin{aligned} \varepsilon '={\varepsilon '_{W}}^\alpha \cdot {\varepsilon '_{T}}^{1-\alpha }, \end{aligned}$$

where $\varepsilon '_{W}$ and $\varepsilon '_{T}$ are the real parts of free water and organic tissue permittivity, $\alpha =TBW\cdot \rho$ is the hydrated rate, $\rho$ is the tissue mass density, and total body water (TBW) is defined as the ratio of the amount of water in the human body divided by the person weight. In particular, for an adult, Eq. (1) becomes

2 $$\begin{aligned} \varepsilon _A'={\varepsilon '_{W}}^{\alpha _A}\cdot {\varepsilon '_{T}}^{1-\alpha _A}\, . \end{aligned}$$

While $\varepsilon '_{W}$ is known from the literature^33^, $\varepsilon '_{T}$ is unknown^13^, but it can be expressed from Eq. (2) as a function of $\varepsilon '_{A}$^34^ and $\varepsilon '_{W}$. The obtained expression for $\varepsilon '$, now depending on $\varepsilon '_{A}$ and $\varepsilon '_{W}$, can be used to compute the real part of the permittivity at a certain age, from the knowledge of the hydrated rate at the same age. The age-dependent complex permittivity can then be computed as

3 $$\begin{aligned} \varepsilon ^*= {\varepsilon '_{W}}^{\frac{\alpha -\alpha _A}{1-\alpha _A}}{\varepsilon '_{A}}^{\frac{1-\alpha }{1-\alpha _A}}\left( 1-j\frac{\varepsilon ''_A}{\varepsilon '_A}\right) \, . \end{aligned}$$

where $\alpha _A$ and $\alpha$ are the hydration rates for an adult and at a specific age, respectively.

This model has been applied to all considered tissues except SC, since the permittivity of this thin uppermost layer is mainly impacted by environmental and physiological conditions rather than by age. The electromagnetic properties of the adult ED, fat, and muscle were set as those of dry skin, noninfiltrated fat, and muscle^34^. The electromagnetic parameters of water and SC are according to Ellison et al.^33^ and Ziskin et al.^18^, respectively. The data for the age-dependent TBW used in this work are shown in Fig. 2b^35,36^. The real permittivity $\varepsilon '$ and conductivity $\sigma$ of the different tissues as a function of age were computed with the Eq. (3) and are shown in Fig. 2c,d at 26 GHz and 60 GHz, respectively.

#### Blood flow

Contrary to the thickness and permittivity, the blood flow influences mainly the thermal diffusion, enhancing the heat dissipation and producing a cooling effect both on the skin surface and in deeper layers^37^. This parameter depends on the on-body location and on age. Measurements reported in the literature demonstrated that this parameter decreases with age^38–41^. We used the following regression based on the measured forearm skin data referring to resting conditions^38^

4 $$\begin{aligned} BF_{D}\left( age\right) =6.033^{-4}-age\cdot 3.55^{-6}\,. \end{aligned}$$

Note that for the IPD below the exposure limits, maximum heating is expected to be limited to several tenths of centigrade, and therefore the blood flow can be considered as almost unchanged during exposure^37^. These values are in accordance with typical blood flow values^18^. Since the forearm is one of the body areas with the lowest blood flow, this corresponds to the worst case condition representing the highest temperature elevation.

### Analytical model

#### Electromagnetic analysis

The electric field is calculated for the stratified model shown in Fig. 1 considering a normally impinging plane wave^42^. This model takes into account multiple reflections occurring at tissue interfaces and the resultant standing waves. The arrows in Fig. 1 indicate, for each layer, the directions of propagation of the incident and reflected waves. The corresponding electric fields, reported for simplicity next to the arrows, are perpendicular to the propagation directions and parallel to the interface (along x direction). An excitation and an observation port were positioned on the top and on the bottom of the structure (along z direction). The total electric field ${\varvec{E}}_i\left( z_i\right)$ is given by the sum of the progressive and regressive fields in the same layer. The total electric field normalized to the incident electric field in the air ($E_0^+$) can be expressed by:

5 $$\begin{aligned} \frac{{\varvec{E}}_i\left( z_i\right) }{{\varvec{E}}_0^+}=\frac{e^{jk_{i}\left( h_{i}-z_{i}\right) }\left( 1+R_ie^{-j2k_{i}\left( h_{i}-z_{i}\right) }\right) }{\displaystyle \prod _{n=0}^{i-1}\frac{1}{1+r_{n}}e^{jk_{n+1}h_{n+1}}\left( 1+r_{n}R_{n+1}e^{-j2k_{n+1}h_{n+1}}\right) }, \end{aligned}$$

where $k_i$ is the wave number in the $i_{th}$ layer, $r_i=\left( n_{i}-n_{i+1}\right) /\left( n_{i}+n_{i+1}\right)$ is the elementary reflection coefficient at the $i_{th}$ interface, depending on $n_i=\sqrt{\varepsilon ^*_i}$ of the two materials separated by the interface and $R_i=E_i^-/E_i^+$ is the total reflection coefficient at the $i_{th}$ interface. The electric field in the muscle can be computed from Eq. (5) considering that there is no reflection from this layer.

SAR is calculated as:

6 $$\begin{aligned} SAR_i=\frac{\sigma _i\left| E_{i_{rms}}\right| ^2}{\rho _i}, \end{aligned}$$

where $E_{i_{rms}}$ is the root mean square value of the electric field, while $\sigma _i$ and $\rho _i$ are the tissue conductivity and density, respectively.

#### Thermal analysis

The bioheat equation^43^ is used to calculate the temperature elevation due to the electromagnetic power absorption in the different layers. The equation, in its monodimensional form and at steady state, is given by

7 $$\begin{aligned} \lambda _i\frac{d^2T_i\left( z_i\right) }{dz_i^2}=B_i\left( T_i(z_i)-T_{blood}\right) -\rho _i SAR_i\left( z_i\right) , \end{aligned}$$

where $T_{blood}={37}\,^{\circ }\hbox {C}$ is the blood temperature, $\lambda _i$ is the thermal conductivity of the $i_{th}$ layer, $B_i=BF_i\rho _bC_b$ is the blood perfusion that depends on the volumetric blood flow rate of the $i_{th}$ layer $BF_i$, on the blood density $\rho _b={1050}\,\hbox {kg m}^{-3}$ and on the heat capacity $C_b={3617}\hbox { J kg}^{{-1}\,\circ }\hbox {C}^{-1}$^44^. The thermal conductivities and densities of the ED, fat, and muscle are taken from Hasgall et al. ^44^. The values of the conductivity and density for the SC layer together with the blood flows for fat and muscle are taken from Christ et al.^19^, while the skin blood flow is from Proctor et al.^38^. Thermal conductivity, mass density, and blood perfusion are reported in Table 1.

**Table 1:** Typical permittivities and thicknesses of tissues, thermal conductivity, mass density and blood perfusion.

Tissue	Typical $\varepsilon ^*$		Typical thickness (mm)	$\lambda _i$ (${\text {W\, m}^{-1\circ }\text {C}^{-1}}$)	${\rho _i (\mathrm{kg\,m}^{-3})}$	$B_i$ (${\mathrm{W\, m}^{-3\circ }}\mathrm{C}^{-1}$)
	26 GHz	60 GHz				
SC	3.62–*j*0.74	3.15–*j*0.50	0.015	0.37	1500	0
Viable epidermis	17.71–*j*16.87	7.98–*j*10.90	1.396	0.37	1109	0
Dermis	17.71–*j*16.87	7.98–*j*10.90	0.060	0.37	1109	$\rho _{b}C_{b}BF_{D}(age)$
Fat	3.76–*j*1.10	3.13–*j*0.84	4	0.21	911	1900
Muscle	25.85–*j*21.84	12.86–*j*15.83	$\infty$	0.49	1090	2550

The temperature distribution in the different layers is obtained analytically solving Eq. (7) with appropriate boundary conditions. For the intermediate layers, we impose the continuity of the temperature and heat flux:

8 $$\begin{aligned} T_i(h_i)=&T_{i+1}(0) \end{aligned}$$

9 $$\begin{aligned} -\lambda _i\frac{dT_i(z_i)}{dz_i}\bigg |_{z_i=h_i}=&-\lambda _{i+1}\frac{dT_{i+1}(z_{i+1})}{dz_{i+1}}\bigg |_{z_{i+1}=0}, \end{aligned}$$

while the boundary conditions for the deepest and the uppermost layer are

10 $$\begin{aligned} T_{last}(\infty )=&T_{blood} \end{aligned}$$

11 $$\begin{aligned} -\frac{dT_1(z_1)}{dz_1}\bigg |_{z_1=0}=&\frac{h}{\lambda _1}\left( T_1(0)-T_{air}\right) , \end{aligned}$$

respectively. The parameter *h* in Eq. (11) is the heat transfer coefficient between air and skin; it is set equal to ${7}{W m}^{-1\circ }{} C^{-1}$^19^. The equations have been solved considering the ambient temperature $T_{air}={20}\,^{\circ }\hbox {C}$.

### Numerical model

#### Electromagnetic analysis

The results computed with the analytical model have been compared with the ones obtained with numerical simulations using the finite element method. The structure has been implemented in COMSOL Multiphysics. The model used for the simulations is the one represented in Fig. 1 with an air layer added on the top of the SC. The thicknesses of the different layers correspond to the ones used in the analytical model, except for the muscle layer that was set to 60 mm. Floquet periodic boundary conditions have been imposed in the directions parallel to the electric field to simulate an infinite structure of stacked dielectric layers, contextually reducing the structure dimension and the computational time. A perfect electric conductor condition is instead applied to the boundaries perpendicular to ${\varvec{E}}$. The structure extends in the *x* and *y* directions for 0.25 mm and is discretized with a tetrahedral mesh (about 50*k* cells in total).

#### Thermal analysis

The steady state temperature distribution with and without the electromagnetic source is computed for the numerical model described in “Electromagnetic analysis” section maintaining the same mesh as that used in the electromagnetic simulations. Adiabatic boundary conditions have been imposed in the *x* and *y* directions and a convective flux with a heat transfer coefficient equal to the one used for the analytical model has been set at the external boundary of the SC. A constant temperature equal to $T_{blood}$ was imposed at the muscle layer termination to reproduce the boundary condition of Eq. (10). Since theoretically $T={37}\,^{\circ }\hbox {C}$ should be imposed at $z=\infty$, the muscle layer thickness was chosen to not alter the temperature distribution.

## Results

### Power density and SAR

The near-surface tissue model presented in Fig. 1 was illuminated by a normally-incident plane wave. Two IPD levels were considered: (1) ${\hbox {IPD}}={10} {\hbox {W m}}^{-2}$ corresponding to the whole-body reference levels in the 6–300 GHz range^5,6^, (2) $IPD =55f_G^{-0.177}$ (30.90 ${\hbox {W m}}^{-2}$ at 26 GHz and 26.65 ${\hbox {W m}}^{-2}$ at 60 GHz) representing the limit for local exposure in the 6–300 GHz range set by ICNIRP.

**Figure 3:**
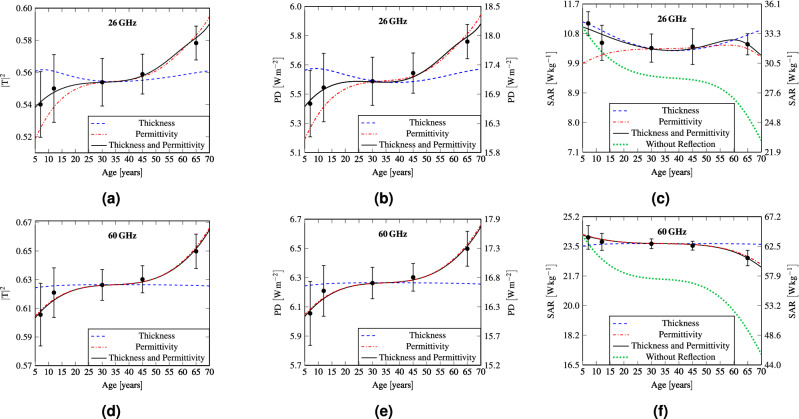
(**a**,**d**) Power transmission coefficient at the air/skin interface, (**b**,**e**) absorbed power density, and (**c**,**f**) peak SAR as a function of skin thickness, tissue permittivity, and combination of the two. The green curve represents the non-reflection condition. The left y axis in (**b**,**c**,**e**,**f**) refers to $IPD ={10}\,{\hbox {W m}}^{-2}$, while the right corresponds to the frequency-dependent IPD limits for local exposure. The bars represent the standard deviation ($P = 0.05$) computed with Monte Carlo simulations, considering a number of trials equal to $10^6$.

First, the effect of the age-dependent variations of the skin thickness and tissue permittivity is studied separately for each parameter in terms of the power transmission coefficient (defined as the ratio between the power transferred to the body and incident power), power density at the interface between the air and the skin (referred as the absorbed power density^5^ or epithelial power density^6^ in exposure guidelines and standards), and SAR, used as a source of heating in bioheat equation (Fig. 3). When only thickness variation is investigated the tissue permittivity is set to its typical value^18,34^ (see Table 1). When permittivity variations are considered, the skin thickness is fixed to 1.396 mm, a typical value for a middle aged man^23^. Second, the effect of age-dependent variation of both parameters is analysed. Similarly to Sasaki et al.^9^, to account for interindividual variations a Monte Carlo analysis^45^ with $10^6$ number of trials was performed for ages with available information about the skin thickness, total body water and body weight (i.e. 7 years, 12 years, 30 years, 45 years, 65 years). A Gaussian probability density function (PDF) with the same mean and standard deviation as those of the corresponding literature data was assigned to the model entries. The Monte Carlo simulation was then carried out changing the parameter values randomly under the assumption of a normal distribution. From the distribution function of the output quantities (power transmission coefficient, absorbed power density and peak SAR ) at each one of the considered ages the mean and uncertainty interval defining a $P=0.05$ were computed. The mean and the uncertainty representing the inter-individual variations are denoted by the black bars in Fig. 3. The results demonstrate that the skin thickness variations in the considered range almost do not change the power transmission coefficient at 60 GHz (Fig. 3d). The changes are more pronounced at 26 GHz (Fig. 3a), in particular $< \ 25$ years.

This can be attributed to a lower $\sigma$ at 26 GHz and thus deeper penetration. In this case, the skin thickness has a stronger influence on the electromagnetic power deposition, modifying the match between the air and the biological tissues. The tissue permittivity is the main parameter impacting the age-dependent variations of the power transmission coefficient at both frequencies. The total variations of the thickness and permittivity result in an increase with age of the power transmission coefficient from 54 to $59\%$ at 26 GHz and from 60.5 to $66\%$ at 60 GHz. This increase is mainly related to the reduction with age of the skin water concentration, that results in a decreasing electromagnetic contrast at the air/skin interface. A plateau is observed between about 25 and 40 years. As expected, the power density follows the same trend as the power transmission coefficient (Fig. 3b,e).

The power absorbed in tissues, characterized by SAR, depends both on the transmission coefficient and tissue conductivity. Contrary to the power density, the peak SAR is almost the double at 60 GHz compared to 26 GHz (Fig. 3c,f), due to a significant difference in conductivity (Fig. 2c,d). It is interesting to note that the trend of SAR is also the opposite in respect to the power density, i.e. the peak SAR decreases with age by $8.76\%$ and $8.44\%$ at 26 GHz and 60 GHz, respectively. This is related to the decreasing with age skin conductivity. Note that at 26 GHz the curve of the peak SAR shows a local maximum at about 60 years that results from the fact that both the considered skin thickness and tissue permittivity contribute to enhance this value. To get a deeper insight into the peak SAR dynamics, it has been computed without accounting for the reflection at the air/skin interface (Fig. 3c,f). To this end, the ED permittivity has been assigned to the air. The curves are normalised to the maximum values of the condition accounting for the reflection at the interface air/skin. At both frequencies, the SAR trend remains almost unchanged, but the curves are steeper than the ones with reflection.

**Figure 4:**
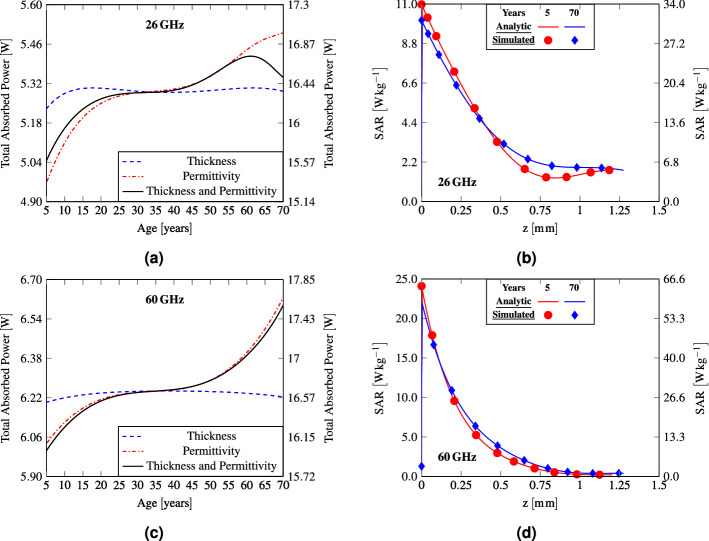
(**a**,**c**) Total absorbed power, (**b**,**d**) SAR distributions in the skin layer as a function of skin thickness, tissue permittivity, and combination of the two. The left y axis refers to IPD equal to 10 ${\hbox {W m}}^{-2}$, while the right y axis corresponds to the frequency dependent IPD limits for local exposure.

The total absorbed power per unitary surface in the skin (SC and ED) as a function of thickness, permittivity and the two parameters combined, at 26 GHz and 60 GHz is represented in Fig. 4a,c, respectively. The total absorbed power in the skin layer does not reflect the trend of the peak SAR. This can be explained through the analytically calculated and numerically computed SAR distribution in the ED layer at two different ages (5 years and 70 years) (Fig. 4b,d). At both frequencies, the peak SAR occurs at the interface between the SC and the viable epidermis. However, since the transmission coefficient is higher and the curves are less steep at 70 years than at 5 years, the corresponding total absorbed electromagnetic power is higher at 70 years, contrary to the peak SAR. At 60 GHz, the curves in Fig. 4c follow the trend of the absorbed/epithelial power density (Fig. 3e). On the contrary, at 26 GHz for elderly people, considering the contextual variation of skin thickness and of tissue permittivity, a local maximum at about 60 years can be noticed similarly to the SAR trend. This is related to the fact that the total absorbed power in the skin layer depends both on complex permittivity and on thickness. Indeed, due to the decrease in water content, the transmission coefficient increases with age, explaining an increase in power absorption, but contextually, skin thickness starts to decrease in old age, thus reducing the portion of tissue in which the power is dissipated. A deeper insight into the total absorbed power in different tissue layers at 26 GHz and 60 GHz for a child (5 years), an adult (35 years), and a senior (70 years) is given in Table 2. Due to the shallow penetration depth at MMW, independently of age and considered frequency, most of the power is absorbed in the skin layer (i.e. more than 90% in ED). However, as a consequence of higher losses at 60 GHz than at 26 GHz, this value ranges from 98.91 to 99.25% at 60 GHz and from 90.51 to 95.46% at 26 GHz. For what concerns the remaining layers, at 60 GHz less than 1% of the power is absorbed in fat and muscle, while at 26 GHz the absorbed power ranges between 3.42 and 6.79% for fat and from 1.18 to 2.54% for muscle. The fraction of the total absorbed power in skin is higher for an adult than for a child or a senior. This could seem in contrast with Fig. 4a,c, since the total absorbed power in skin, intended as its absolute value, is higher at 70 years than at 35 years for both the analysed frequencies. However, at 35 years SAR is steeper and skin is thicker than at 70 years, so that even if the power transmitted to the tissues is higher at older ages, the fraction of the one dissipated in skin is lower. When comparing a child with a senior the absorbed power in skin is higher at younger ages. In fact, even if the skin thickness is almost the same and the power transmission coefficient is higher at 70 years than at 5, the lower SAR gradient in the z direction at older ages is responsible for the lower absorption as percentage of the electromagnetic power in the skin layer.

**Table 2:** Percentage of total absorbed power in SC, ED, fat and muscle at 26 GHz and 60 GHz for a child (5 years), an adult (35 years) and a senior (70 years).

Tissue	Child (5 years)		Adult (35 years)		Senior (70 years)	
	26 GHz (%)	60 GHz (%)	26 GHz (%)	60 GHz (%)	26 GHz (%)	60 GHz (%)
SC	0.13	0.28	0.14	0.30	0.16	0.33
ED	93.61	99.25	95.46	99.41	90.51	98.91
Fat	5.08	0.44	3.42	0.27	6.79	0.69
Muscle	1.18	0.03	0.98	0.02	2.54	0.07

**Table 3:** Temperature rise variation with age (when considering the effect of blood flow, permittivity and all the contribution contextually) and maximal temperature rise (absolute value).

Frequency ( GHz)	${\Delta T}$	${\Delta T}$	${\Delta T}$	${T_{max}} ({}\,^{\circ }\hbox {C})$	${T_{max}} ({}\,^{\circ }\hbox {C})$
	Blood flow (%)	Permittivity (%)	All (%)	$\mathbf {IPD={10}\,{\hbox {W m}}^{-2}}$	$\mathbf {IPD=55f}_\mathbf{G}^{-0.177}\,{\hbox {W m}}^{-2}$
26	2.28	12.87	10.82	0.172	0.532
60	3.35	9.78	13.87	0.202	0.538

### Heating

In this section, we study the effect of ageing on the temperature elevation. In this case, in addition to the skin thickness and tissue permittivity, also the variations of the blood flow were taken into account.

**Figure 5:**
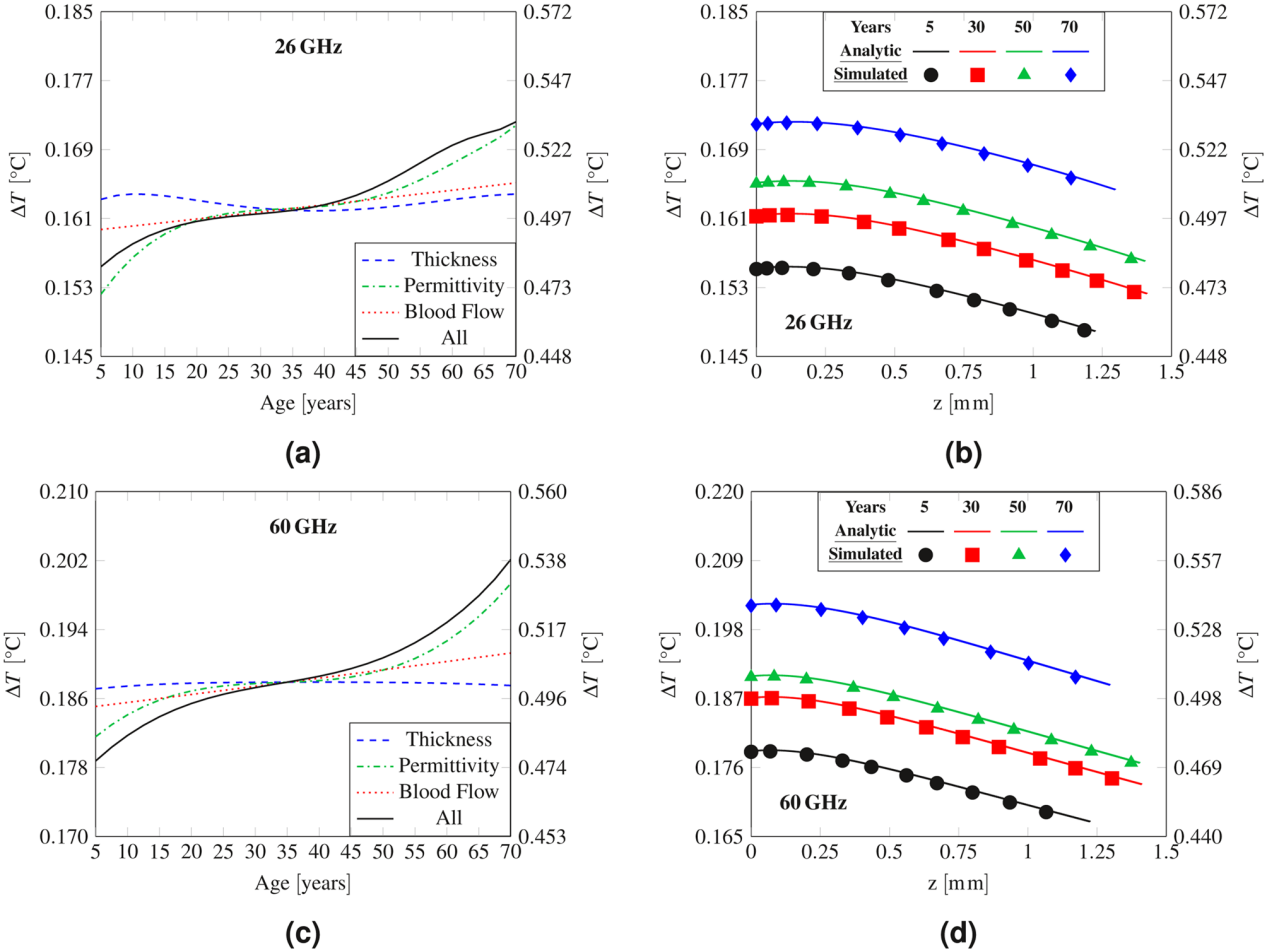
(**a**,**c**) Peak steady-state temperature elevation in skin as a function of age, (**b**,**d**) temperature elevation as a function of depth. The left y axis refers to the case of IPD equal to 10 ${\hbox {W m}}^{-2}$, while the right y axis corresponds to the frequency-dependent IPD limits for local exposure.

The steady-state temperature rise in the skin layer considering variations with age of the skin thickness, tissue permittivity, and blood flow are reported in Fig. 5a,c at 26 GHz and 60 GHz, respectively.

The results demonstrate that the skin thickness variation has a negligible effect, as for the electromagnetic quantities. Permittivity and blood flow have instead a more pronounced impact on the temperature elevation and both cause a higher temperature rise at older ages. In particular, at 26 GHz the maximum temperature rise with age in skin increases by $12.87\%$, when only the permittivity contribution is considered and by $2.28\%$ for the blood flow (see Table 3). Instead, at 60 GHz the increase corresponds to $9.78\%$ and $3.35\%$ for permittivity and blood flow, respectively. Considering the combined variation of skin thickness, permittivity and blood flow the increase with age is $10.82\%$ and $13.07\%$ at 26 GHz and 60 GHz, respectively. A decrease of the blood flow with age implicates a reduced ability to dissipate heat and results in a higher temperature rise, while the effect of permittivity, in particular at 60 GHz, reflects the trend of the total absorbed power in the skin layer (Fig. 4c). However, contrary to the total absorbed power at 26 GHz, considering the contextual variation of thickness, permittivity and blood flow, the maximum temperature increases monotonically with age. In fact, the effect of the local maximum at 60 years in Fig. 4a is reduced, because of the skin thickness and the decrease in blood flow at ages above 60 years, which determinate a stronger temperature rise after 60 years.

Note that the maximum temperature elevation occurs at 70 years for both frequencies, and for $IPD={10}\,{\hbox {W m}}^{-2}$ it is higher at ${60} \,\hbox {GHz} \,({0.202}\,^{\circ }\hbox {C})$ than at ${26} \,\hbox {GHz}\, ({0.172}\,^{\circ }\hbox {C})$. On the contrary, considering the frequency dependent limits of IPD, the maximal temperature is only slightly different at the two frequencies $({0.532}\,^{\circ }\hbox {C}$ at 26  GHz and ${0.538}\,^{\circ }\hbox {C}$ at 60  GHz).

Figure 5b,d represent the heating distribution in the skin (SC and ED) obtained with the analytical model and in full-wave simulations, incorporating the thickness, permittivity, and blood flow variations. All the curves demonstrate similar dynamics and highlight the worst condition for aged people due to the effect of permittivity and blood flow reduction.

## Discussion and conclusion

During the ageing process electromagnetic properties of human tissues vary influencing electromagnetic power absorption and resulting heating in the human body. Previous studies investigated the effect of age at lower microwave frequencies (up to 5.6 GHz), demonstrating that the exposure levels in children can exceed those in adults^14,16,17^.

In this study, we used measured anatomical data available in the literature to model the impact of age variations on the dissipated power and resulting heating at 26 GHz and 60 GHz, frequencies upcoming for 5G and future generations of wireless communication systems. Fundamental electromagnetic and thermal quantities, such as the power transmission coefficient, the absorbed/epithelial power density, the peak SAR, and the temperature elevation were analytically calculated and numerically computed using a multilayer model of near surface biological tissues. To analyse the impact of the tissue ageing, the variations of skin thickness and tissue permittivity were considered. Furthermore, the blood flow was also taken into account to assess heating. A normally impinging plane wave was used as a source. Note that the biological aspects and considerations related to potential age-dependent health effects are out of the scope of this electromagnetic and thermal dosimetry study. The near surface planar multilayer model used in this work has been validated in former dosimetry studies at millimeter waves^9–12^. The investigation of the interface roughness and continuity constitutes one of the perspectives of this study. At the considered frequencies, most of the electromagnetic power is dissipated in the skin layer (more than 90%). Therefore the fat and muscle layers have a negligible impact from the electromagnetic viewpoint (less than 10%) and are mainly considered to characterize the thermal diffusion. Also the SC with typical thicknesses (${10}{-}{15}\,\upmu \hbox {m}$) has an almost negligible effect on the power deposition ^19^. The interindividual variations were taken into account through a statistical analysis considering typical variations of the input parameters available in the literature.

The dosimetric quantities of interest were computed considering both the whole body IPD limits (10 ${\hbox {W m}}^{-2}$) and the frequency dependent ones (30.90 ${\hbox {W m}}^{-2}$ at 26 GHz and 26.65 ${\hbox {W m}}^{-2}$ at 60 GHz). These limits are set by the guidelines^5,6^ to prevent from possible health effects based on *in vitro*, *in vivo* and epidemiological studies^5,6^. They are defined applying a safety factor of 10 for occupational and 50 for general public exposure to the threshold values for health effect. Exposure at IPD levels corresponding to the specified limits is then expected to protect both children and adults. However, at MMW no data concerning possible differences in terms of the electromagnetic power absorption and heating as a function of ageing are available, and the aim of this work is to fill this gap.

Our results demonstrate that, at both 26 GHz and 60 GHz, the ageing effect produces variations comparable or, for specific ages (e.g. the power density at 12 years), slightly higher than the variations related to the inter-individual differences. In particular, the effect of the skin thickness on the electromagnetic power deposition and temperature rise is almost negligible, while permittivity variations have a stronger impact. Due to the reduction of the water content with age the real part of permittivity $\varepsilon '$ and the conductivity $\sigma$ decrease with age. As a consequence, the power transmission coefficient and consequently the power density at the interface air/skin increase with age. At 26 GHz the transmitted power increases from 54% at 5 years to 59% at 70 years, while at 60 GHz it increases from 60.5% at 5 years to 66% at 70 years. This increase is more pronounced during the youth and after 50 years, with a plateau between roughly 25 years and 50 years. It is interesting to note that, contrary to the transmission coefficient and the absorbed power density, the peak SAR decreases with age as a consequence of the conductivity decrement. While the transmission coefficient and absorbed power density are comparable at the two frequencies, the SAR at 60 GHz is almost double compared to the one at 26 GHz, due to the different conductivity values at the two frequencies. Independently of age, the most of power is absorbed in ED. At 60 GHz the fraction of power absorbed in the remaining layers does not exceed 0.7%, while at 26 GHz it reaches 6.8% in fat and 2.5% in muscle. Contrary to peak SAR, in ED the fraction of power absorbed in this layer does not decrease with age and is higher in adults than in children and seniors. The difference between seniors and children is explained by the fact that at 5 years and 70 years the skin thickness is almost the same, while the conductivity is higher in children. In adults, instead, the conductivity is intermediate between the children and the senior ones, but the thickness is more elevated, thus explaining the higher fraction of power absorbed in the ED layer. However, even if for seniors the fraction of power absorbed in ED and the peak SAR are the lowest, since the power transmission coefficient is higher compared to lower ages, the total absorbed power absolute value is higher than at lower ages.

The maximal steady-state heating increases with age, and reaches its maximum at 70 years. For older ages the temperature increase is enhanced as a consequence of the higher absorbed power and reduced blood flow that limits the heat dissipation in skin. For $IPD={10}\,{\hbox {W m}}^{-2}$, the maximum temperature elevation ranges from ${0.16}\,^{\circ }\hbox {C}$ (5 years) to ${0.17}\,^{\circ }\hbox {C}$ (70 years) at 26 GHz and from ${0.18}\,^{\circ }\hbox {C}$ (5 years) to ${0.20}\,^{\circ }\hbox {C}$ (70 years) at 60 GHz. Considering the local frequency-dependent limits, the maximum heating is of ${0.532}\,^{\circ }\hbox {C}$ at 26 GHz and of ${0.538}\,^{\circ }\hbox {C}$ at 60 GHz. These values remain well below the environmental temperature fluctuations to which skin is exposed in natural conditions.

Overall our results demonstrate that, considering a planar tissue model illuminated by a plane wave, the age-dependent variations of the electromagnetic power deposition and resulting heating in the near-surface tissues are limited to about 10–15%. These variations are of the same order as inter-individual differences. Furthermore, they are much lower compared to the safety margins applied to exposure limits (i.e. 50 for general public and 10 for occupational exposure^5,6^). Validation of these conclusions on more realistic models (e.g. user terminal close to a head model) is out of the scope of this study and constitutes one of its perspectives.
