# Publisher Correction: Age-dependence of electromagnetic power and heat deposition in near-surface tissues in emerging 5G bands

**DOI:** 10.1038/s41598-021-92059-5

**Published:** 2021-06-10

**Authors:** Giulia Sacco, Stefano Pisa, Maxim Zhadobov

**Affiliations:** 1 Department of Information Engineering, Electronics and Telecommunications, Sapienza University of Rome, 00184 Rome, Italy; 2 Univ Rennes, CNRS, IETR (Institut d’Électronique et des Technologies du numéRique) UMR 6164, 35000 Rennes, France

Correction to: *Scientific Reports* 10.1038/s41598-021-82458-z, published online 17 February 2021

The original version of this Article contained typographical errors.

In Figure 5b,d the solid lines representing the analytic results did not display correctly.

The original Figure 5 and accompanying legend appear below.

**Figure 5:**
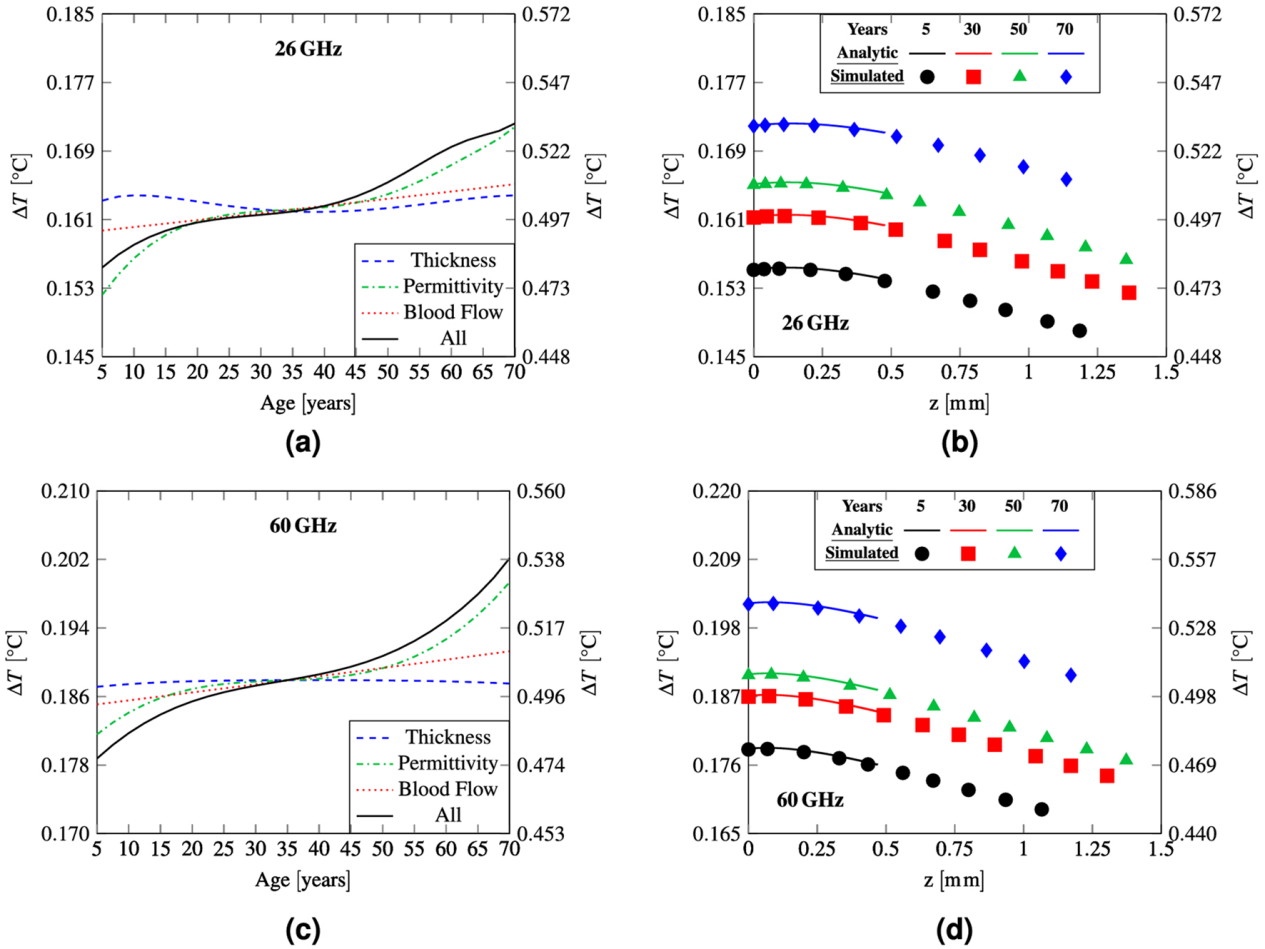
(**a**,**c**) Peak steady-state temperature elevation in skin as a function of age, (**b**,**d**) temperature elevation as a function of depth. The left y axis refers to the case of IPD equal to 10 W m^−2^, while the right y axis corresponds to the frequency-dependent IPD limits for local exposure.

The original Article has been corrected.

